# 5-Chloro-1-(4-methyl­phenyl­sulfon­yl)-1*H*-indole

**DOI:** 10.1107/S1600536812046466

**Published:** 2012-11-17

**Authors:** Mohammad Hassam, Vincent J. Smith

**Affiliations:** a Department of Chemistry and Polymer Science, University of Stellenbosch, Private Bag X1, Matieland 7602, South Africa

## Abstract

In the title compound, C_15_H_12_ClNO_2_S, the indole ring is essentially planar (r.m.s. deviation = 0.0107 Å) and makes a dihedral angle of 85.01 (6)° with the benzene ring. In the crystal, three C—H⋯O hydrogen bonds result in a hydrogen-bonded spiral running parallel to the *c* axis.

## Related literature

For background to the use of indoles as scaffolds in the synthesis of HIV-agents, see: Hassam *et al.* (2012 ▶). For the crystal structure of a closely related compound, see: Beddoes *et al.* (1986 ▶).
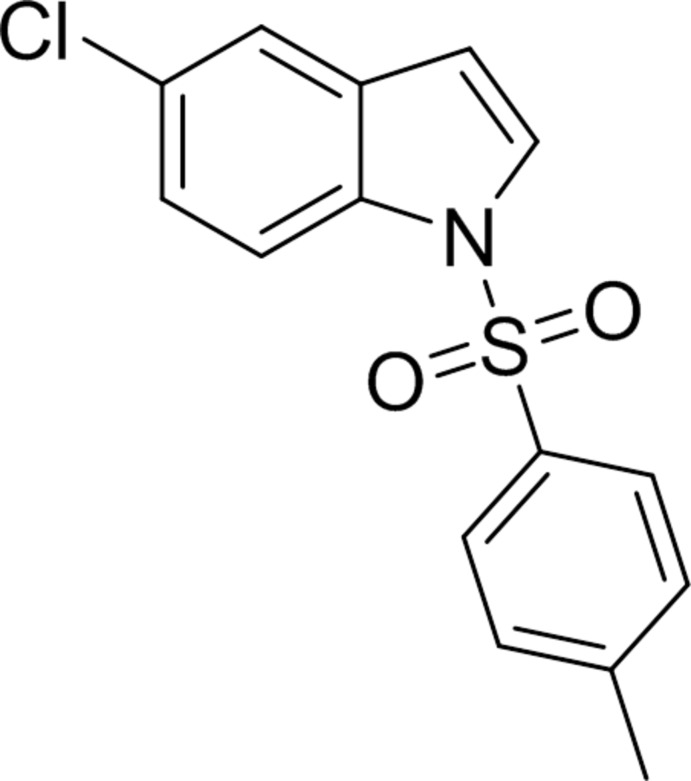


## Experimental

### 

#### Crystal data

C_15_H_12_ClNO_2_S
*M* *_r_* = 305.77
Tetragonal, 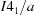

*a* = 26.991 (7) Å
*c* = 7.8345 (19) Å
*V* = 5708 (2) Å^3^
*Z* = 16
Mo *K*α radiation
μ = 0.41 mm^−1^
*T* = 111 K
0.1 × 0.1 × 0.01 mm

#### Data collection

Bruker APEXII CCD diffractometer
Absorption correction: multi-scan (*SADABS*; Bruker, 2009 ▶) *T*_min_ = 0.950, *T*_max_ = 0.968
17940 measured reflections
3565 independent reflections
2760 reflections with *I* > 2σ(*I*)
*R*_int_ = 0.038

#### Refinement

*R*[*F* ^2^ > 2σ(*F* ^2^)] = 0.039
*wR*(*F* ^2^) = 0.101
*S* = 1.06
3565 reflections
182 parameters
H-atom parameters constrained
Δρ_max_ = 0.31 e Å^−3^
Δρ_min_ = −0.36 e Å^−3^

### 

Data collection: *APEX2* (Bruker, 2009 ▶); cell refinement: *SAINT* (Bruker, 2009 ▶); data reduction: *SAINT*; program(s) used to solve structure: *SHELXS97* (Sheldrick, 2008 ▶); program(s) used to refine structure: *SHELXL97* (Sheldrick, 2008 ▶); molecular graphics: *X-SEED* (Barbour, 2001 ▶; Atwood & Barbour, 2003 ▶); software used to prepare material for publication: *X-SEED*.

## Supplementary Material

Crystal structure: contains datablock(s) I, global. DOI: 10.1107/S1600536812046466/pv2597sup1.cif

Structure factors: contains datablock(s) I. DOI: 10.1107/S1600536812046466/pv2597Isup2.hkl

Supplementary material file. DOI: 10.1107/S1600536812046466/pv2597Isup3.cml

Additional supplementary materials:  crystallographic information; 3D view; checkCIF report

## Figures and Tables

**Table 1:** Hydrogen-bond geometry (Å, °)

*D*—H⋯*A*	*D*—H	H⋯*A*	*D*⋯*A*	*D*—H⋯*A*
C2—H2⋯O2^i^	0.95	2.54	3.327 (2)	140
C4—H4⋯O1^ii^	0.95	2.49	3.192 (2)	131
C14—H14⋯O1^iii^	0.95	2.59	3.333 (2)	135

Symmetry codes: (i) 
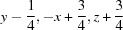
; (ii) 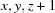; (iii) 
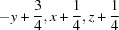
.

**Fig. 1.**
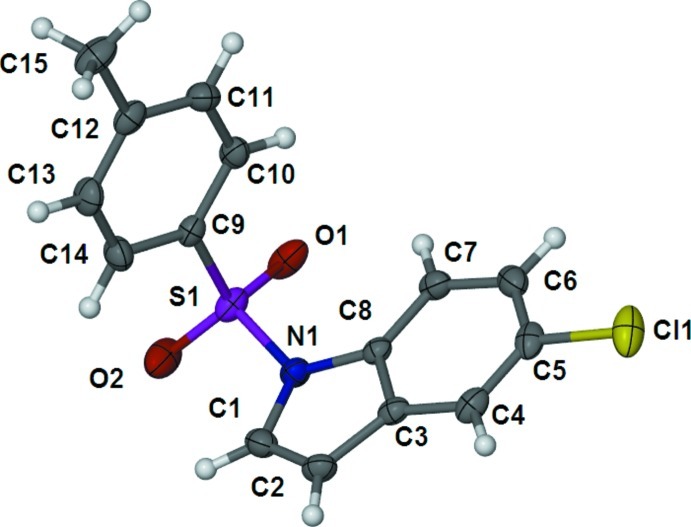
The molecular structure of the title compound with the atom numbering scheme. Displacement ellipsoids are drawn at the 50% probability level. H atoms are presented as small spheres of arbitrary radius.

**Fig. 2.**
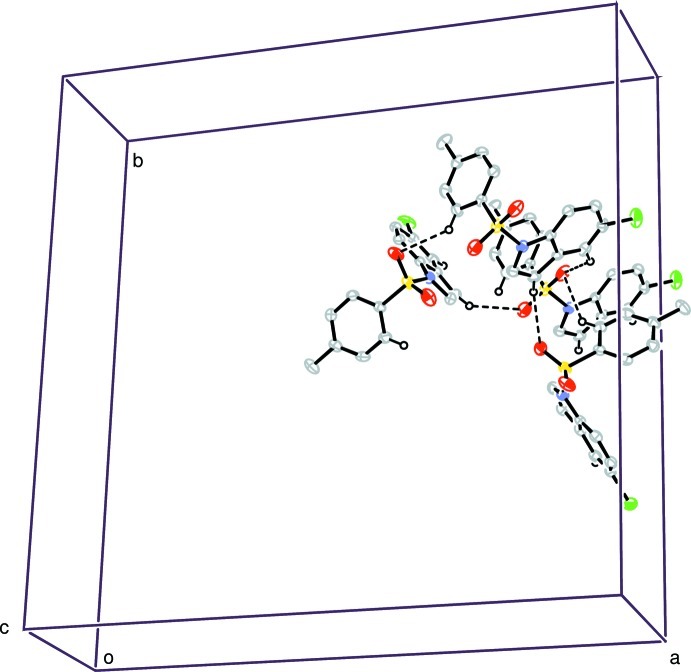
A view of the C—-H···O hydrogen bonds (dotted lines) in the crystal structure of the title compound. H atoms non-participating in hydrogen- bonding were omitted for clarity.

## supplementary crystallographic information

### Comment

5-Chloroindole is frequently employed as a building block in the synthesis of various biologically active molecules (Hassam *et al.*, 2012). For this communication, tosylation of 5-chloroindole was performed by deprotonation of the indole with sodium hydride, followed by the addition of tosyl chloride.

In the title molecule (Fig. 1), the indole ring is essentially planar (rmsd = 0.0107 Å) with Cl1 lying in its plane (deviation 0.008 (2) Å). The dihedral angle between the mean planes of the indole and benzene rings is 85.01 (6)°. The bond distances and angles in the title compound agree very well with the corresponding bond distances and angles reported for 1-phenylsulfonyl-indole (Beddoes *et al.*, 1986). There are three C—H···O hydrogen bonds which connect four symmetry related molecules into a hydrogen bonded spiral that runs parallel to the *c*-axis (Table 1 and Fig. 2).

### Experimental

Sodium hydride (0.325 g, 14.1 mmol) was added to a solution of 5-chloroindole (1.00 g, 6.59 mmol) in dry THF (50 ml) at 273.15 K (ice bath). The reaction mixture was stirred at the same temperature for 10 min, followed by addition of tosyl chloride (4-methyl-benzene-1-sulfonyl chloride, 1.88 g, 9.86 mmol). The reaction mixture was then stirred at 273.15 K for 2 h. Water (25 ml) was added to the flask and the mixture was extracted with diethyl ether (2 *x* 25 ml). The organic layer was washed with brine (25 ml) and dried over anhydrous sodium sulfate. Solvent was removed under vacuum and the resulting residue was recrystallized from hexane and dichloromethane (4:1) to obtain the title compound as a colourless crystalline material (1.61 g, 80%).

### Refinement

The H atoms were positioned geometrically and refined using a riding model, with C—H = 0.95 and 0.98 Å, for aryl and methyl H-atoms, respectively. The *U*_iso_(H) were allowed at 1.5*U*_eq_(methyl C) or 1.2*U*_eq_(aryl C).

### Crystal data

**Table:**

C_15_H_12_ClNO_2_S	*D*_x_ = 1.423 Mg m^−^^3^
*M**_r_* = 305.77	Melting point: 383 K
Tetragonal, *I*4_1_/*a*	Mo *K*α radiation, λ = 0.71073 Å
Hall symbol: -I 4ad	Cell parameters from 3900 reflections
*a* = 26.991 (7) Å	θ = 3.0–25.8°
*c* = 7.8345 (19) Å	µ = 0.41 mm^−^^1^
*V* = 5708 (2) Å^3^	*T* = 111 K
*Z* = 16	Plate, colourless
*F*(000) = 2528	0.1 × 0.1 × 0.01 mm

### Data collection

**Table:**

Bruker APEXII CCD diffractometer	3565 independent reflections
Radiation source: fine-focus sealed tube, Bruker SMART APEXII	2760 reflections with *I* > 2σ(*I*)
Graphite monochromator	*R*_int_ = 0.038
φ and ω scans	θ_max_ = 28.9°, θ_min_ = 2.1°
Absorption correction: multi-scan (*SADABS*; Bruker, 2009)	*h* = −36→26
*T*_min_ = 0.950, *T*_max_ = 0.968	*k* = −36→30
17940 measured reflections	*l* = −10→9

### Refinement

**Table:**

Refinement on *F*^2^	Primary atom site location: structure-invariant direct methods
Least-squares matrix: full	Secondary atom site location: difference Fourier map
*R*[*F*^2^ > 2σ(*F*^2^)] = 0.039	Hydrogen site location: inferred from neighbouring sites
*wR*(*F*^2^) = 0.101	H-atom parameters constrained
*S* = 1.06	*w* = 1/[σ^2^(*F*_o_^2^) + (0.0395*P*)^2^ + 5.9559*P*] where *P* = (*F*_o_^2^ + 2*F*_c_^2^)/3
3565 reflections	(Δ/σ)_max_ = 0.001
182 parameters	Δρ_max_ = 0.31 e Å^−^^3^
0 restraints	Δρ_min_ = −0.36 e Å^−^^3^

### Special details

**Table:**

Geometry. All e.s.d.'s (except the e.s.d. in the dihedral angle between two l.s. planes) are estimated using the full covariance matrix. The cell e.s.d.'s are taken into account individually in the estimation of e.s.d.'s in distances, angles and torsion angles; correlations between e.s.d.'s in cell parameters are only used when they are defined by crystal symmetry. An approximate (isotropic) treatment of cell e.s.d.'s is used for estimating e.s.d.'s involving l.s. planes.
Refinement. Refinement of *F*^2^ against ALL reflections. The weighted *R*-factor *wR* and goodness of fit *S* are based on *F*^2^, conventional *R*-factors *R* are based on *F*, with *F* set to zero for negative *F*^2^. The threshold expression of *F*^2^ > σ(*F*^2^) is used only for calculating *R*-factors(gt) *etc*. and is not relevant to the choice of reflections for refinement. *R*-factors based on *F*^2^ are statistically about twice as large as those based on *F*, and *R*- factors based on ALL data will be even larger.

### Fractional atomic coordinates and isotropic or equivalent isotropic displacement parameters (Å^2^)

**Table:**

	*x*	*y*	*z*	*U*_iso_*/*U*_eq_	
S1	0.309589 (17)	0.612848 (17)	0.38622 (6)	0.02904 (13)	
Cl1	0.494357 (19)	0.67701 (2)	0.99440 (8)	0.04639 (17)	
C9	0.27756 (6)	0.66717 (7)	0.4379 (2)	0.0244 (4)	
N1	0.33623 (5)	0.59487 (5)	0.56768 (19)	0.0257 (3)	
O2	0.27557 (6)	0.57429 (5)	0.34641 (19)	0.0423 (4)	
C1	0.31014 (7)	0.56870 (7)	0.6945 (3)	0.0306 (4)	
H1	0.2805	0.5505	0.6762	0.037*	
C5	0.44798 (7)	0.65358 (7)	0.8611 (3)	0.0304 (4)	
O1	0.34919 (5)	0.62485 (6)	0.27270 (16)	0.0384 (3)	
C4	0.41247 (7)	0.62301 (7)	0.9286 (2)	0.0299 (4)	
H4	0.4129	0.6143	1.0461	0.036*	
C8	0.37656 (6)	0.61872 (6)	0.6465 (2)	0.0223 (3)	
C6	0.44862 (7)	0.66693 (7)	0.6895 (3)	0.0321 (4)	
H6	0.4741	0.6879	0.6477	0.039*	
C7	0.41247 (7)	0.64987 (7)	0.5795 (2)	0.0282 (4)	
H7	0.4122	0.6591	0.4625	0.034*	
C3	0.37571 (6)	0.60502 (6)	0.8196 (2)	0.0245 (4)	
C11	0.27459 (7)	0.75507 (7)	0.4642 (3)	0.0324 (4)	
H11	0.2898	0.7865	0.4496	0.039*	
C10	0.30006 (7)	0.71282 (7)	0.4162 (2)	0.0290 (4)	
H10	0.3324	0.7151	0.3692	0.035*	
C2	0.33347 (7)	0.57340 (7)	0.8452 (2)	0.0306 (4)	
H2	0.3238	0.5585	0.9499	0.037*	
C12	0.22732 (7)	0.75243 (7)	0.5330 (2)	0.0298 (4)	
C14	0.23055 (7)	0.66336 (7)	0.5066 (3)	0.0325 (4)	
H14	0.2155	0.6319	0.5215	0.039*	
C13	0.20586 (7)	0.70613 (8)	0.5533 (3)	0.0343 (4)	
H13	0.1735	0.7038	0.6002	0.041*	
C15	0.19970 (8)	0.79851 (8)	0.5824 (3)	0.0417 (5)	
H15A	0.1849	0.7940	0.6956	0.063*	
H16B	0.1735	0.8050	0.4987	0.063*	
H17C	0.2227	0.8266	0.5851	0.063*	

### Atomic displacement parameters (Å^2^)

**Table:**

	*U*^11^	*U*^22^	*U*^33^	*U*^12^	*U*^13^	*U*^23^
S1	0.0320 (3)	0.0321 (3)	0.0230 (2)	0.00953 (18)	−0.00349 (18)	−0.00451 (18)
Cl1	0.0310 (3)	0.0522 (3)	0.0560 (3)	0.0064 (2)	−0.0073 (2)	−0.0294 (3)
C9	0.0226 (8)	0.0297 (9)	0.0209 (8)	0.0049 (7)	−0.0012 (7)	−0.0012 (7)
N1	0.0264 (8)	0.0276 (8)	0.0231 (7)	0.0022 (6)	−0.0005 (6)	0.0019 (6)
O2	0.0480 (9)	0.0340 (8)	0.0448 (9)	0.0065 (6)	−0.0175 (7)	−0.0127 (7)
C1	0.0291 (10)	0.0247 (9)	0.0382 (10)	−0.0007 (7)	0.0041 (8)	0.0034 (8)
C5	0.0243 (9)	0.0296 (9)	0.0375 (10)	0.0075 (7)	−0.0022 (8)	−0.0120 (8)
O1	0.0414 (8)	0.0520 (9)	0.0218 (6)	0.0217 (7)	0.0058 (6)	0.0028 (6)
C4	0.0309 (10)	0.0356 (10)	0.0230 (9)	0.0116 (8)	0.0002 (7)	−0.0038 (8)
C8	0.0226 (8)	0.0221 (8)	0.0224 (8)	0.0063 (6)	0.0019 (7)	0.0009 (6)
C6	0.0243 (9)	0.0261 (9)	0.0460 (11)	0.0023 (7)	0.0051 (8)	−0.0002 (8)
C7	0.0268 (9)	0.0296 (9)	0.0283 (9)	0.0054 (7)	0.0062 (7)	0.0056 (7)
C3	0.0262 (9)	0.0243 (8)	0.0230 (8)	0.0079 (7)	0.0039 (7)	0.0019 (7)
C11	0.0338 (10)	0.0267 (9)	0.0368 (11)	0.0006 (8)	0.0011 (8)	0.0051 (8)
C10	0.0234 (9)	0.0347 (10)	0.0287 (9)	0.0025 (7)	0.0048 (7)	0.0051 (8)
C2	0.0327 (10)	0.0302 (10)	0.0288 (10)	0.0028 (7)	0.0048 (8)	0.0083 (8)
C12	0.0312 (10)	0.0360 (10)	0.0221 (9)	0.0096 (8)	−0.0027 (7)	−0.0014 (8)
C14	0.0240 (9)	0.0331 (10)	0.0405 (11)	−0.0029 (7)	0.0030 (8)	−0.0014 (8)
C13	0.0219 (9)	0.0430 (11)	0.0379 (11)	0.0040 (8)	0.0075 (8)	−0.0015 (9)
C15	0.0483 (13)	0.0416 (12)	0.0352 (11)	0.0223 (10)	−0.0008 (9)	−0.0042 (9)

### Geometric parameters (Å, º)

**Table:**

S1—O2	1.4224 (15)	C6—C7	1.381 (3)
S1—O1	1.4278 (15)	C6—H6	0.9500
S1—N1	1.6654 (15)	C7—H7	0.9500
S1—C9	1.7496 (18)	C3—C2	1.438 (3)
Cl1—C5	1.7487 (19)	C11—C10	1.383 (3)
C9—C14	1.382 (2)	C11—C12	1.387 (3)
C9—C10	1.384 (3)	C11—H11	0.9500
N1—C8	1.407 (2)	C10—H10	0.9500
N1—C1	1.408 (2)	C2—H2	0.9500
C1—C2	1.345 (3)	C12—C13	1.387 (3)
C1—H1	0.9500	C12—C15	1.501 (3)
C5—C4	1.371 (3)	C14—C13	1.382 (3)
C5—C6	1.392 (3)	C14—H14	0.9500
C4—C3	1.396 (3)	C13—H13	0.9500
C4—H4	0.9500	C15—H15A	0.9800
C8—C7	1.386 (2)	C15—H16B	0.9800
C8—C3	1.406 (2)	C15—H17C	0.9800
			
O2—S1—O1	120.84 (9)	C8—C7—H7	121.3
O2—S1—N1	104.64 (8)	C4—C3—C8	119.13 (17)
O1—S1—N1	105.94 (8)	C4—C3—C2	133.19 (17)
O2—S1—C9	110.18 (9)	C8—C3—C2	107.68 (16)
O1—S1—C9	108.90 (9)	C10—C11—C12	121.37 (18)
N1—S1—C9	105.07 (8)	C10—C11—H11	119.3
C14—C9—C10	121.13 (17)	C12—C11—H11	119.3
C14—C9—S1	118.77 (14)	C11—C10—C9	118.83 (17)
C10—C9—S1	120.05 (13)	C11—C10—H10	120.6
C8—N1—C1	107.88 (14)	C9—C10—H10	120.6
C8—N1—S1	125.11 (12)	C1—C2—C3	107.74 (16)
C1—N1—S1	122.18 (13)	C1—C2—H2	126.1
C2—C1—N1	109.77 (17)	C3—C2—H2	126.1
C2—C1—H1	125.1	C13—C12—C11	118.36 (17)
N1—C1—H1	125.1	C13—C12—C15	120.65 (18)
C4—C5—C6	122.48 (18)	C11—C12—C15	120.98 (19)
C4—C5—Cl1	119.19 (15)	C9—C14—C13	118.91 (18)
C6—C5—Cl1	118.33 (15)	C9—C14—H14	120.5
C5—C4—C3	118.06 (17)	C13—C14—H14	120.5
C5—C4—H4	121.0	C14—C13—C12	121.40 (17)
C3—C4—H4	121.0	C14—C13—H13	119.3
C7—C8—C3	122.42 (17)	C12—C13—H13	119.3
C7—C8—N1	130.71 (16)	C12—C15—H15A	109.5
C3—C8—N1	106.86 (15)	C12—C15—H16B	109.5
C7—C6—C5	120.51 (18)	H15A—C15—H16B	109.5
C7—C6—H6	119.7	C12—C15—H17C	109.5
C5—C6—H6	119.7	H15A—C15—H17C	109.5
C6—C7—C8	117.39 (17)	H16B—C15—H17C	109.5
C6—C7—H7	121.3		
			
O2—S1—C9—C14	27.82 (18)	C5—C6—C7—C8	−1.0 (3)
O1—S1—C9—C14	162.50 (15)	C3—C8—C7—C6	0.9 (3)
N1—S1—C9—C14	−84.38 (16)	N1—C8—C7—C6	−178.52 (16)
O2—S1—C9—C10	−154.75 (15)	C5—C4—C3—C8	0.1 (2)
O1—S1—C9—C10	−20.06 (17)	C5—C4—C3—C2	−179.33 (18)
N1—S1—C9—C10	93.06 (16)	C7—C8—C3—C4	−0.5 (3)
O2—S1—N1—C8	172.54 (14)	N1—C8—C3—C4	179.06 (15)
O1—S1—N1—C8	43.81 (16)	C7—C8—C3—C2	179.10 (16)
C9—S1—N1—C8	−71.38 (15)	N1—C8—C3—C2	−1.36 (18)
O2—S1—N1—C1	−35.12 (16)	C12—C11—C10—C9	0.0 (3)
O1—S1—N1—C1	−163.85 (14)	C14—C9—C10—C11	−0.2 (3)
C9—S1—N1—C1	80.95 (15)	S1—C9—C10—C11	−177.58 (14)
C8—N1—C1—C2	−2.7 (2)	N1—C1—C2—C3	1.8 (2)
S1—N1—C1—C2	−159.19 (13)	C4—C3—C2—C1	179.22 (19)
C6—C5—C4—C3	−0.2 (3)	C8—C3—C2—C1	−0.3 (2)
Cl1—C5—C4—C3	179.67 (13)	C10—C11—C12—C13	0.1 (3)
C1—N1—C8—C7	−178.07 (17)	C10—C11—C12—C15	−179.14 (18)
S1—N1—C8—C7	−22.5 (3)	C10—C9—C14—C13	0.3 (3)
C1—N1—C8—C3	2.45 (18)	S1—C9—C14—C13	177.70 (15)
S1—N1—C8—C3	158.06 (12)	C9—C14—C13—C12	−0.2 (3)
C4—C5—C6—C7	0.7 (3)	C11—C12—C13—C14	0.0 (3)
Cl1—C5—C6—C7	−179.22 (14)	C15—C12—C13—C14	179.24 (19)

### Hydrogen-bond geometry (Å, º)

**Table:**

*D*—H···*A*	*D*—H	H···*A*	*D*···*A*	*D*—H···*A*
C2—H2···O2^i^	0.95	2.54	3.327 (2)	140
C4—H4···O1^ii^	0.95	2.49	3.192 (2)	131
C14—H14···O1^iii^	0.95	2.59	3.333 (2)	135
C7—H7···O1	0.95	2.44	3.025 (2)	120
C10—H10···O1	0.95	2.59	2.943 (2)	102

Symmetry codes: (i) *y*−1/4, −*x*+3/4, *z*+3/4; (ii) *x*, *y*, *z*+1; (iii) −*y*+3/4, *x*+1/4, *z*+1/4.
